# Decreased cerebrospinal fluid TNFRSF8 (sCD30) confirmed as a biomarker of Huntington’s disease progression

**DOI:** 10.1007/s00415-026-14112-5

**Published:** 2026-09-12

**Authors:** Helga Maria Grétarsdóttir, Janet L. Cunningham, Annica Rasmusson, Joachim Burman, Kim Kultima, Martin Paucar, Per Svenningsson, Radu Constantinescu, Valter Niemelä

**Affiliations:** 1 Department of Medical Sciences, Neurology, Uppsala University, Uppsala, Sweden; 2 Department of Neurology, Uppsala University Hospital, Uppsala, Sweden; 3 Department of Medical Sciences, Clinical Psychiatry, Uppsala University, Uppsala, Sweden; 4 Department of Psychiatry, Uppsala University Hospital, Uppsala, Sweden; 5 Department of Clinical Neuroscience, Karolinska Institute, Stockholm, Sweden; 6 Department of Neurology, Karolinska University Hospital, Uppsala, Sweden; 7 Department of Neuroscience and Physiology, Clinical Neuroscience, The Sahlgrenska Academy, University of Gothenburg, Gothenburg, Sweden

**Keywords:** Huntington’s disease, Proteomics, Biomarker, Neuroinflammation, TNFRSF8, SCD30

## Abstract

**Background:**

In Huntington’s disease (HD), clinical heterogeneity in age at onset and disease progression poses demands on precision medicine approaches. Biofluid biomarkers are needed to monitor disease progression, but may also provide insights into pathogenic mechanisms and support the development of disease-modifying therapies (DMTs).

**Objective:**

To identify dysregulated cerebrospinal fluid (CSF) proteins in *HTT* gene expansion carriers (HDGECs), and to evaluate their relationship with disease progression.

**Method:**

CSF was collected from a discovery and a validation cohort, both comprising HDGECs and neurologically unaffected controls (HCs). A total of 734 proteins were quantified using a high-sensitive Proximity Extension Assay (PEA) (Olink Neuro Explore panel 1 and 2). Protein association with disease progression was evaluated using the composite Unified HD Rating Scale (cUHDRS). Disease stage was classified according to the HD Integrated Staging System (HD-ISS).

**Results:**

In total, samples included HDGECs (*n*=61), HCs (*n*=54), and longitudinal HDGEC samples (*n*=21). Sixteen proteins were nominally dysregulated in HDGECs vs. HCs. Most pronounced was a twofold decrease of TNFRSF8 (sCD30) in HDGECs, and longitudinally declining levels were found. TNFRSF8 correlated significantly with cUHDRS, after adjustment for age, sex, and CAG. Levels of TNFRSF8 were similarly reduced in the validation cohort, and correlated with Total Functional Capacity (TFC).

**Conclusion:**

TNFRSF8 recently emerged as a biomarker of neuroimmune dysfunction in HD. The results indicate and confirm that it is highly dysregulated in HDGECs and also tracks clinical disease progression. This pathway may be a promising potential target for DMTs.

**Supplementary Information:**

The online version contains supplementary material available at https://doi.org/10.1007/s00415-026-14112-5.

## Background

Huntington’s disease (HD) is an autosomal dominant neurodegenerative disorder caused by an expansion of the CAG-trinucleotide repeat of the huntingtin (*HTT*) gene [1]. The toxic effect of the expanded CAG tract causes dysfunction and eventual loss of vulnerable neurons, especially in the striatum and basal ganglia, subsequently involving the cortex [2]. HD clinically manifests as a triad of motor symptoms (including chorea, dystonia and later bradykinesia/rigidity), cognitive decline, and psychiatric or behavioral disturbances, which often precedes the onset of motor signs [2, 3].

Although CAG repeat length shows a strong association with age at onset and disease severity, it explains up to 70% of the variability in onset age and disease progression [4]. This suggests that additional factors, including multiple genetic modifiers, contribute to the marked heterogeneity in disease onset and progression [5]. This clinical heterogeneity complicates patient selection for clinical trials and highlights the need for biomarkers that accurately reflect current disease activity and prognosis. Clinical assessment is particularly challenging during the premanifest phase, as subclinical and subtle symptoms may evolve over decades, and the transition to manifest disease is often difficult to define clinically [6]. Biomarkers may also support precision medicine approaches, including identification of therapeutic targets, trial enrichment, and monitoring treatment response [7].

Among the most promising biomarkers identified to date are neurofilament light chain (NfL), a non-specific marker of neuronal injury that strongly correlates with disease activity in HD [8, 9], and proenkephalin, which has recently emerged as a biomarker with greater specificity for striatal degeneration [10, 11]. While NfL is a well-established indicator of neuroaxonal damage, it does not directly capture the biological pathways that may influence disease progression, such as immune activation and neuroinflammatory responses [12, 13].

Previous biomarker discovery approaches have largely relied on targeted analyses of candidate proteins using enzyme-linked immunosorbent assay (ELISA) and mass spectrometry (LC–MS/MS). More recently, newly developed multiplex proximity extension assays (PEA) offer enhanced sensitivity with minimal sample volume requirements, allowing simultaneous quantification of a large number of proteins, including those present in low abundance [14].

Using a high-sensitivity multiplex assay approach, we aimed to identify novel dysregulated cerebrospinal fluid (CSF) proteins in *HTT* gene expansion carriers (HDGECs) compared with neurologically unaffected controls, and to evaluate associations to disease progression.

## Method

### Participants and study design

This cross-sectional observational study analyzed clinical and cerebrospinal fluid (CSF) biomarker data from adults (> 18 years of age) carrying a pathogenic *HTT* CAG repeat expansion (≥ 35 repeats). Participants were offered an optional follow-up visit for repeat clinical assessment and sample collection, at least 1 year after the initial visit.

This study was based on CSF samples collected within the Uppsala Huntington’s disease CSF (Uppsala HDCSF) study, an ongoing longitudinal study of individuals with Huntington’s disease and neurologically unaffected controls, (hereafter referred to as “controls”), as previously described [9]. No subjects with other significant medical or neurological conditions were enrolled. Current immunosuppressive treatment was also considered an exclusion criterion. This approach was used to minimize the risk that underlying neurological or systemic disease, or treatment-related effects, could influence CSF biomarker measurements.

Discovery cohort: Participants, including HDGECs and controls, were recruited through the regional HD clinic at Uppsala University Hospital. Additional CSF control samples were obtained from individuals undergoing clinical evaluation for neurological symptoms at the Department of Neurology at Uppsala University Hospital. The participants had presented with either headache or transient sensory symptoms but had normal findings on standard neurological evaluation, including lumbar puncture, and where applicable, neuroradiological imaging. All participants were assessed by a neurologists and cleared of neurological disease prior to inclusion, with the clinical investigations subsequently reviewed by the study authors to verify eligibility.

Validation cohort: This study encompassed a validation cohort including HDGECs and controls which were recruited in parallel from HD clinics from the Karolinska Institute, Stockholm, and Sahlgrenska University Hospital, Gothenburg.

All participants signed a written informed consent before enrollment. The study protocol was approved by the Regional Ethics Review board of Uppsala (Dnr 2012/274), and samples from Karolinska Institute were included under approval Dnr 2012/2224–32/4. The study procedure was in accordance with the Declaration of Helsinki.

### Clinical and cognitive assessment

Premanifest HDGECs were defined as individuals who did not meet the criteria for manifest HD on the Unified HD Rating Scale (UHDRS) motor examination, corresponding to a diagnostic confidence level (DCL) below 4, while manifest HD was defined by DCL of 4.

Clinical assessment was conducted using the composite UHDRS (cUHDRS), that incorporates motor, cognitive, and functional components into a novel scoring algorithm designed to capture overall disease impairment. There is no fixed theoretical range; however, a higher cUHDRS score indicates better combined motor, cognitive, and functional status [15]. cUHDRS includes the following assessment scales: Total Motor Score (TMS; scores: 0–124), Symbol Digit Modalities Test (SDMT; range ≥ 0–110), Stroop Word Test (SWT; range ≥ 0–120) and the Total Functional Capacity (TFC; range 0–13) scale.

Disease stage was determined according to the Huntington’s Disease Integrated Staging System (HD-ISS), which classifies HDGECs into four stages (0–3) based on CAG repeat length, clinical measures (SDMT and TMS), TFC and neuroimaging markers of striatal atrophy [16]. As neuroimaging data were not available in the present study, CSF neurofilament light (NfL) levels were used to approximate Stage 1 classification [17]. An age-adjusted threshold for ISS 1 was applied, defined as the levels above 95th percentile of control participants [18]. Of note, participants were eligible for inclusion even if they carried an expanded *HTT* CAG repeat of ≥35 repeats due to a previously defined inclusion criteria used for the Uppsala HDCSF study. Consequently, participants with CAG repeat lengths of 35–39 could be included in the study even if this does not match formal HD-ISS stage classification, which requires ≥40 CAG repeats.

Genetic disease burden was quantified using the normalized CAG–Age-Product (CAP100) Score. This measure reflects cumulative exposure to the expanded *HTT* allele and proximity to clinical onset [19], where a CAP score of 100 corresponds to the predicted time of motor onset [20].

### Sample collection and handling

CSF was collected by lumbar puncture according to standardized protocol for procedure, materials, and handing. Clinical assessments were conducted on the same day as CSF collection in the vast majority of participants, with only a minority performed within 3 months of the sampling date. CSF was collected in low-binding polypropylene tubes, centrifuged at 4 °C at 1300 g for 10 min, aliquoted, and stored at −80 °C. All samples remained frozen and underwent a single thaw cycle prior to analysis.

### CSF proteomic analysis

CSF proteins were quantified using a Proximity Extension Assay (PEA). A total of 734 assays were included using the commercially available Olink Explore Neurology 1 and 2 panels, (Olink Proteomics AB, Uppsala, Sweden) at the Department of Affinity Proteomics Uppsala NGI-Uppsala, SNP&SEQ Technology Platform.

The Olink explore platform yields protein level readout from massive parallel sequencing reported as normalized protein expression (NPX) values, a log^2^-transformed relative measure [10]. Each sample was analyzed using the Olink Explore platform with built-in internal quality controls to ensure data validity and assay performance. These included incubation, extension, and amplification controls incorporated into each well to monitor critical reaction steps, as well as plate controls used for normalization across plates and experimental runs.

In addition, three external control samples were included to evaluate reproducibility and technical variability across plates. Owing to the limited number of external controls, a formal calculation of the limit of detection (LOD) was not performed.

Raw count data were quality controlled according to the manufacturer guidelines and normalized using Olink’s standard intensity normalization procedure, which corrects for technical variation between samples and plates [21]. The samples were run on two plates, with a coefficient of variation of <4.7%. Assays not meeting Olink quality control criteria were excluded. In addition, a median count threshold of >150 (among samples with counts >10) was applied to select assays with the strongest signals. Normalized protein expression (NPX) values were used for all downstream statistical analyses.

Neurofilament light protein (NfL) was measured using two platforms where the NfL levels from Olink were mainly used in the analyses. Exclusively used for HD-ISS classification, NfL concentrations were measured using a Quanterix NF-light Single Molecule Array (Simoa) assay (Quanterix), following established protocols [22] at the Department of Medical Sciences, Uppsala University.

### Statistical analysis

As protein levels were log^2^-transformed, parametric statistical methods were applied unless otherwise specified. According to a pre-specified analysis plan, group differences in CSF biomarker concentrations between HD-ISS stages 1–3 and HCs were assessed using linear regression adjusted for age and sex. Participants classified as HD-ISS stage 0 were excluded from this analysis as they were expected to be far from disease onset and therefore less likely to show detectable proteomic changes. All HDGECs (HD-ISS 0–3) from the discovery cohort, including repeated measures, were included in a linear mixed-effects model to evaluate the association between protein levels and cUHDRS, adjusted for CAG repeat length, age, and sex. The estimate corresponds to the log^2^ fold change for a one unit decrease in cUHDRS. For correlation analyses, Pearson’s correlation coefficients between protein levels and cUHDRS were calculated using cross-sectional data from all gene expansion carriers only.

To account for multiple testing, the significance threshold was adjusted based on the estimated number of independent protein groups (*n* = 205), identified using principal component analysis (PCA) and correlation clustering. Using a significance level of α = 0.05, the corrected *p* value threshold was set at 0.00024 (0.05/205), to reduce false-positive findings.

Potential confounders including age, sex, BMI, CAG repeat number, CAP100 score, as well as storage time and collection site, were assessed by including these as covariates in simple linear regression models with the same threshold for significance (*p* = 0.00024).

## Results

### Discovery cohort, clinical data

In the discovery cohort, a total of 82 participants were enrolled between 2012 and 2025 (Table 1). Among manifest HDGECs (*n*=22), the majority were in advanced disease stage (HD-ISS stage 3). Of the 20 premanifest HDGECs, the majority were in HD-ISS stage 1. Two participants had a CAG repeat length below 40 (CAG_n_ = 39 in both cases) but were nevertheless classified according to the HD-ISS framework, as this staging system was introduced after the initiation of the sample collection utilized in present study.

**Table 1:** Baseline Clinical Data of the Discovery Cohort

	Controls (*n*=40)	Premanifest HDGEC (*n*=20)	Manifest HDGEC (*n*=22)
Repeated sample *n*= (mean days, SD)	0	9 (648.4, 440.7)	12 (997.4, 721.1)
Sex, women (%)	24 (60%)	12 (60%)	9 (41%)
Age mean (SD)	40.2 (14.3)	34.4 (9.6)	53.3 (13.0)
CAG mean (SD)		43.3(3.4)	43.1 (2.8)
CAP100 mean (SD)		67.5 (14.1)	105.7 (13.8)
Years to onset mean (SD)		17.7 (8.6)	NA
Shoulson stage ***mean (SD)			1.82 (0.8)
HD-ISS, *n* per stage (0/1/2/3)		(6/10/4/0)	(0/1/2/19)
cUHDRS mean (SD)		15.8 (1.5)	7.8 (4.4)
TFC		13 (0)	9.5 (2.8)
TMS		1.6 (1.4)	28.2 (14.7)
SDMT		45.5 (11.3)	21.8 (9.8)
SWT		86.3 (14.7)	53.1 (22.3)
NfL (pg/ml) mean (SD)	470.3 (198)	1387.2 (904.6)	3802.5 (1397.5)

Table entries show mean or n (%) and standard deviation (SD)

**Langbehn et.al 5-year risk of onset [23]

***Shoulson stage

*HDGEC* Huntington’s disease gene expansion carriers, *CAG* length of Cytosine−Adenine−Guanine (CAG) repeat expansion in the Huntingtin (HTT) gene, *CAP100* CAG−Age−Product Score, *HD*−*ISS* Huntington’s Disease Integrated Staging System, *TFC* Total Functional Capacity, *TMS* Total Motor Score, *SDMT* Symbol Digit Modalities Test, *SWT* Stroop Word Test, *NfL* Neurofilament light protein

Repeat CSF samples were collected from 21 individuals, of which 12 were manifest and 9 premanifest HDGECs, with a follow-up interval between 1 and 7.6 years. One participant phenoconverted to manifest HD between the first and second study visits, and was included in the premanifest HDGEC group but data from both visits were used where applicable.

Participants with two visits had in some cases incomplete clinical data for one of the visits, and in this case, data from the other visit were used. Still, some HDGECs had missing data limited to no BMI (*n*=5), or no cUHDRS (*n*=4). These participants were included in analyses that did not involve the missing variables.

Forty controls were enrolled, including fourteen neurologically healthy donors and twenty-six neurologically assessed individuals without evidence of neurological disease.

### Validation cohort

Nineteen HDGECs spanning from HD-ISS stages 1–3 were included in the analysis (with mean values on TFC score of 9, and CAP100 of 107) together with fourteen controls. cUHDRS data were not assessed in this cohort.

### Quality control of assays and associations to confounders

Of 734 protein assays measured across 136 samples, 20 assays did not meet the Olink quality control criteria for batch release and were excluded from further analysis. Subsequent analysis focused on the 442 assays with a median count of at least 150.

To evaluate potential confounding effects on CSF protein levels, we assessed interactions between protein abundance among and the following variables: age, sex, CAG repeat length, CAP100, and BMI, in all HDGECs from the discovery cohort (*n*=42). According to simple linear regression analysis (with *p-cutoff* <0.00024), significant associations were identified for age (96 proteins), sex (1 protein: CD99), CAG-repeat length (1 protein; TNFRSF8), CAP100 (51 proteins). No significant associations were observed for BMI. Linear regression analysis performed in the discovery cohort control group (*n*=40) identified 10 proteins associated with age, 0 proteins associated with BMI, 2 proteins associated with sex (CD99, CCL19), and 0 protein associated with storage time with *p-cutoff* 0.00024.

### Discovery cohort; proteomic dysregulation in HDGECs

The discovery cohort participants classified as HD-ISS stage 0 (*n*=6) were excluded from statistical analysis to increase sensitivity for detecting disease-associated proteomic changes. A complete list of proteins differentiating HDGECs from controls is found in Supplemental Table 1.

**Fig. 1:**
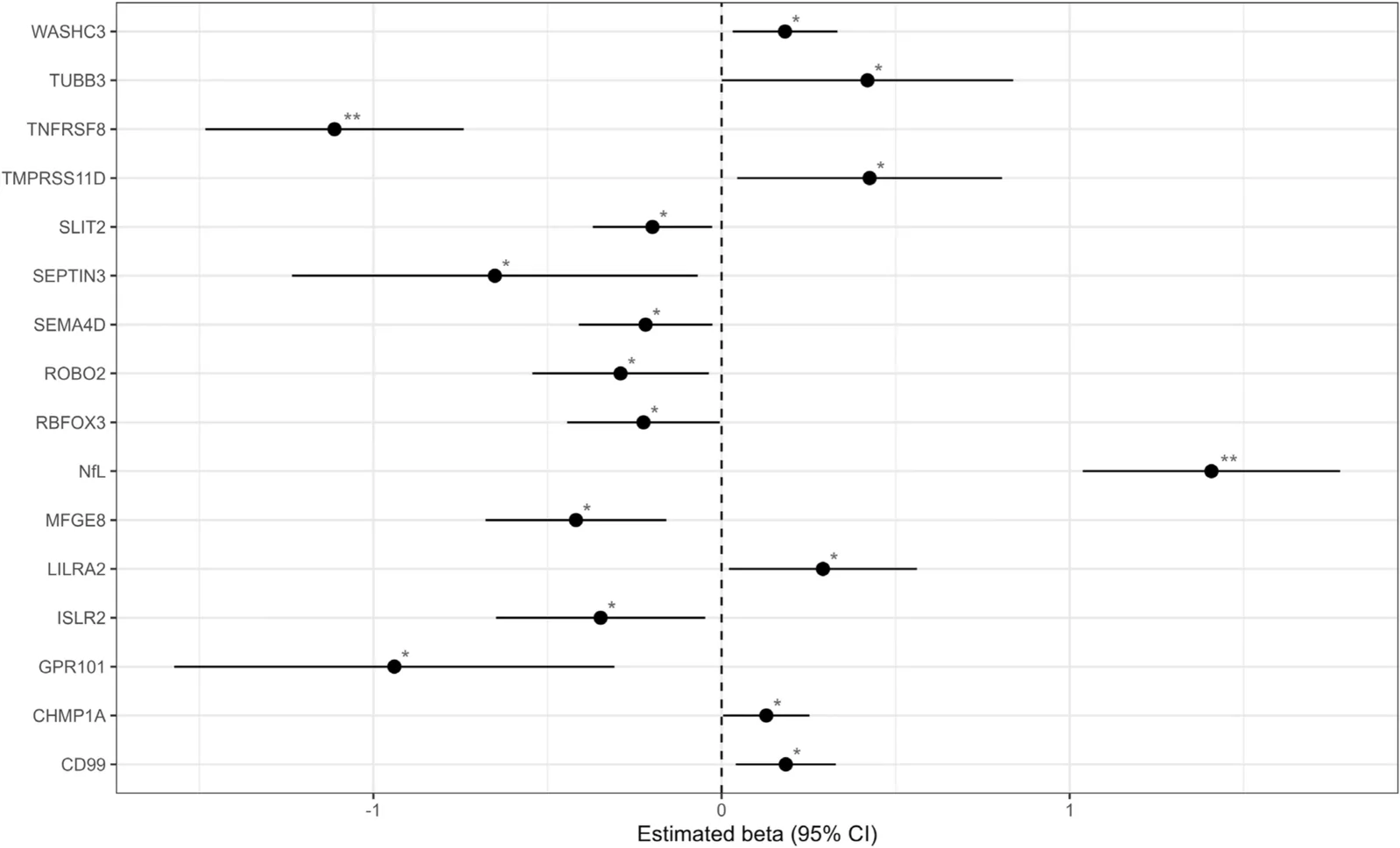
Proteins with the strongest difference between HD-ISS 1–3 and controls of the discovery cohort. 14 proteins had nominally significant (*) alterations in HD-ISS 1-3 compared with controls after adjustment for age and sex. Only NfL and TNFRSF8 were significantly altered after adjustment for multiple testing (**).* NPX* Normalized Protein expression units,* a log*^2^ transformed relative measure,* CI* confidence interval,* P* p value

Comparing the relative protein concentration between HD-ISS 1–3 (*n*= 36) and controls (*n*= 40) using a regression model (Fig. 1), including age and sex as confounders, resulted in a total of 16 dysregulated proteins, 14 of which had nominal *p* values <0.05. Roughly half (7/16) of these were increased.

After multiple-testing correction, only NfL and TNFRSF8 levels remained significantly different between participants with HD-ISS stages 1–3 and controls. The reduction of TNFRSF8 remained significant after adjustment for age (*p*= 4.2 × 10^−8^, beta= −1.13, CI −1.50, −0.76) and after adjustment for age and sex (*p*= 7.6 × 10^−8^, beta= −1.11, CI −1.48, −0.74). The difference for NfL also remained significant after adjustment for age and sex. There was a weak but nominally significant correlation between TNFRSF8 and NfL (*r* = −0.35, *p* = 0.02) among all HDGECs.

Among the additional dysregulated proteins, both MFGE8 and GPR101 showed nominally significant reduction in HD-ISS stages 1–3 participants compared with controls in analyses adjusted for age alone (MFGE8: *p*=0.002, beta= −0.42, CI −0.67, −0.16; GPR101: *p*=0.004, beta= −0.93, CI −1.56, −0.31), as well as in analyses adjusted for both age and sex (MFGE8: *p*=0.002, beta= − 0.42, CI −0.68, −0.16; GPR101: *p*=0.004, beta= − 0.94, CI −1.57, −0.31).

**Fig. 2:**
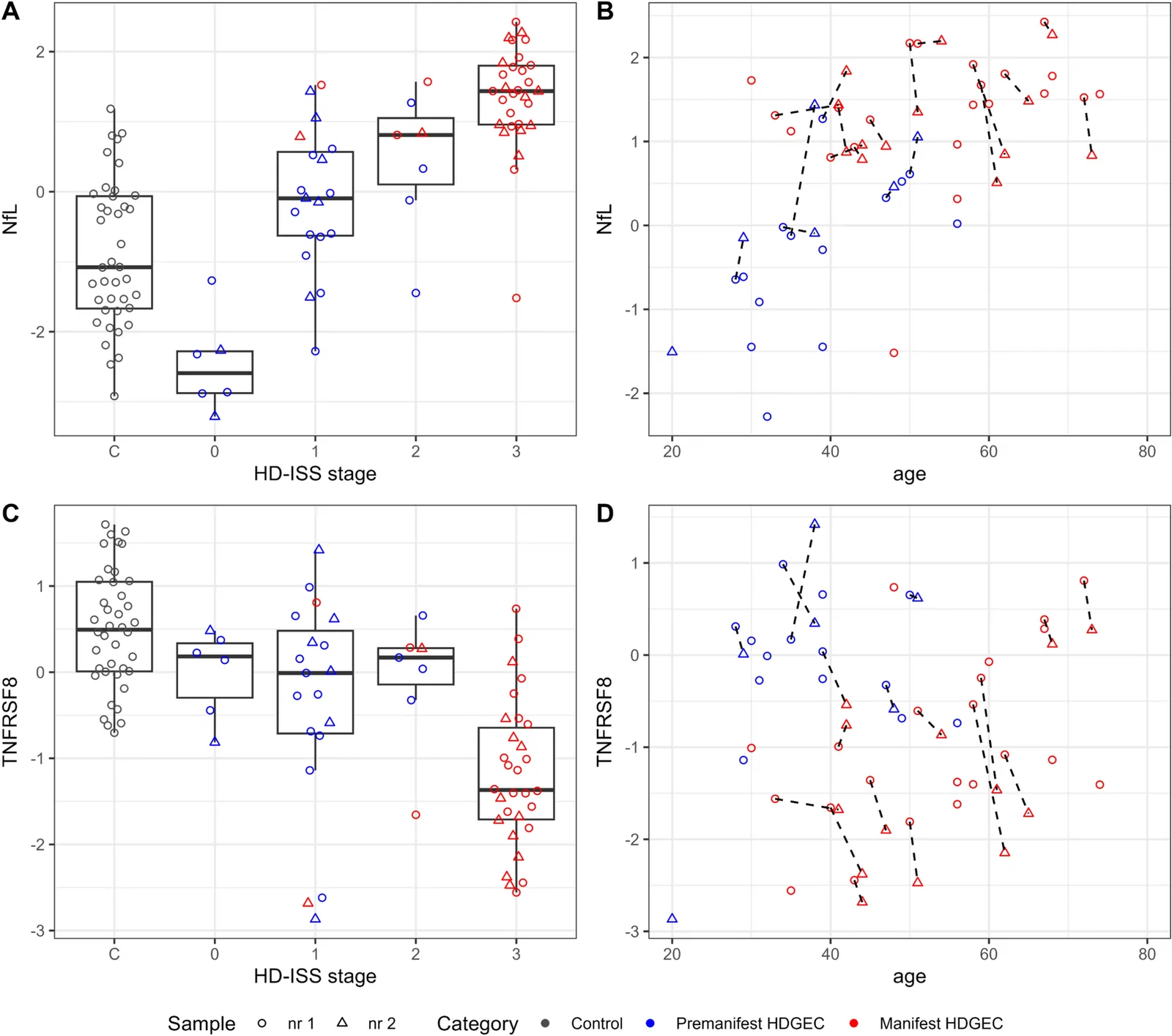
Discovery cohort; NPX levels of NfL and TNFRSF8 distributed across HD-ISS stages, and their longitudinal measurements. The levels of NfL** A**, and TNFRSF8** C** were significantly altered in HD-ISS 1-3 vs. controls of the Discovery cohort. In the longitudinal analysis from 21 gene expansion carriers, no difference in NfL was seen (*p*>0.9), but** D** a reduction of TNFRSF8 over time (*p* = 0.017) was found.* C* Healthy controls,* HD-ISS* HD-Integrated Staging System stages 0-3,* NPX* relative protein concentration units,* a log*^2^ transformed relative measure. Age in years,* Sample nr* Indicates whether patient’s sample was collected at 1^st^ or 2^nd^ visit,* PreHD* premanifest HD gene expansion carriers,* HD manifest* HD gene expansion carrier

Longitudinal analysis of TNFRSF8 levels was carried out among 21 HDGECs with a total of 42 repeated samples (Fig. 2), and a mean interval of 648.4 days between samples (range 347–1377) for premanifest and 997.4 days (range 322–2786) for manifest participants between visits. A linear mixed-effects model indicated that TNFRSF8 levels decreased over time (*p* = 0.017, beta= −0.11, CI − 0.21, −0.02). On average, TNFRSF8 decreased by 0.11 NPX units per year. An interaction term between HD category (manifest HDGEC vs. premanifest HDGEC) and time was introduced in the model, but was not statistically significant (*p* = 0.066). Furthermore, in the interaction model, the rate of annual change was pronounced in the manifest HDGEC group (beta −0.14, CI −0.24, − 0.04), whereas no change was noted in the premanifest HDGEC group (beta +0.07, CI −0.13, +0.27).

Using identical modeling for NfL resulted in no change over time (*n*=21, beta − 0.006, *p*>0.9). Similarly, the interaction term between category and time was trending toward but not formally significant in the model (*p*=0.059). A non-significant trend toward increasing values among premanifest HDGECs (beta +0.16 CI −0.06, +0.39), and unchanged or slightly decreasing values in the manifest HDGECs (beta −0.08 CI −0.19, +0.03) was noted.

### Proteins associated with symptom severity in the discovery cohort

In order to test associations between protein levels and cUHDRS scores, data from all HDGECs including their repeated visits (where available) were included in a linear mixed-effects model (Table 2). For the complete list of proteins and associations with cUHDRS, see Supplemental Table 2.

**Table 2:** Discovery cohort; top ten protein associated with cUHDRS

	No adjustment			Adjusted for age			Adjusted for age, CAG, and sex			Pearson correlation	
Assay	Estimate	CI	P	Estimate	CI	p	Estimate	CI	p	r	p
NfL	−0.13	−0.19, −0.07	4.0 × 10^−5^	−0.07	−0.13, −0.01	0.032	−0.03	−0.09, 0.03	0.372	−0.69	2.1 × 10^−6^
SFRP1	−0.13	−0.19, −0.06	2.3 × 10^−4^	−0.08	−0.15, −0.01	0.033	−0.09	−0.17, −0.00	0.044	−0.61	4.1 × 10^−5^
WASHC3	−0.04	−0.06, −0.02	3.0 × 10^−4^	−0.02	−0.04, 0.00	0.089	−0.02	−0.04, 0.00	0.115	−0.51	0.001
TNFRSF8	0.08	0.04, 0.13	3.8 × 10^−4^	0.10	0.05, 0.16	1.5 × 10^−4^	0.06	0.01, 0.11	0.017	0.40	0.013
PRDX1	−0.07	−0.11, −0.03	6.4 × 10−4	−0.05	−0.10, −0.00	0,048	−0.04	−0.09, 0.02	0,171	−0.51	0,001
PSME1	−0.03	−0.05, −0.01	0,002	−0.02	−0.04, −0.00	0,042	−0.03	−0.05, −0.00	0,028	−0.46	0,004
DHRS4L2	−0.03	−0.05, −0.01	0,005	−0.01	−0.04, 0.01	0,238	−0.02	−0.05, 0.01	0,134	−0.58	1.3 × 10−4
EFNA1	−0.05	−0.08, −0.02	0,005	−0.02	−0.06, 0.02	0,293	−0.03	−0.08, 0.01	0,108	−0.56	2.2 × 10−4
SPAG1	−0.07	−0.12, −0.02	0,008	−0.05	−0.11, 0.01	0,123	−0.08	−0.15, −0.01	0,027	−0.29	0,08
NDRG1	−0.04	−0.08, −0.01	0,013	−0.02	−0.06, 0.02	0,345	−0.02	−0.06, 0.03	0,452	−0.52	7.9 × 10−4

Only the proteins with the strongest associations are shown. Negative values indicate that higher protein levels are associated with worse clinical function, while positive values indicate the opposite

*CAG* length of Cytosine−Adenine−Guanine (CAG) repeat expansion in the Huntingtin (HTT) gene

Sixty-two proteins were nominally associated with cUHDRS without adjustment for confounders, of which only two (IFT20, TNFRSF8) decreased with symptom progression.

The proteins NfL, SFRP1, WASHC3 and TNFRSF8 showed the strongest associations with cUHDRS (*p*= 4.0 × 10^−5^, *p*= 2.3 × 10^−4^, *p*=3.0 × 10^−4^, *p*= 3.8 × 10^−4^ resp.) (Fig. 2, Table 2). In the unadjusted model, proteins NfL and SFRP1 showed negative association with cUHDRS (beta= −0.13 for both) and significant p values, indicating that higher concentrations were linked to worse clinical performance. WASHC3 had a weaker negative association (beta= −0.04), while TNFRSF8 displayed a positive association (beta= 0.08).

After adjusting for age, the effect sizes of all four previously mentioned proteins were reduced but remained directionally consistent, and the proteins NfL, SFRP1 had nominally significant associations. After adjustment for age, sex, and CAG, TNFRSF8 was the only nominally significant protein (beta= 0.06, *p*=0.017, CI 0.01, 0.11).

TNFRFS8 was nominally associated with CAP100 score (*p*=0.007) and demonstrated the strongest association with CAG repeat length among all 442 proteins included in the panel (*r*= −0.59, *p*= 3.53× 10^−8^). There were no significant associations between TNFRSF8 and age (*p*=0.167) or sex (*p*= 0.092).

Pearson correlations supported the above associations as the proteins (NfL, SFRP1, WASHC3, and TNFRSF8) displayed moderate-to-strong correlations with cUHDRS (rho = −0.69, −0.61, −0.51; and 0.40, respectively).

Predicted time to onset was assessed in all premanifest HD from the discovery cohort (*n*=20), who were estimated to be an average, 17.9 years from disease onset according to the Langbehn model [23]. Simple linear regression modeling found that, after adjustment for multiplicity, only NfL remained significantly associated with predicted disease onset, with higher levels observed in participants closer to disease onset (*p*=2.7 × 10^−4^, beta= −0.096). Eight proteins; MRPL46, NLGN1, GIGYF2, LRFN2, MRPL28, CACNA1C, MAG, and GSTT2B, also had nominal association.

For TNFRSF8, a non-significant trend was observed for the association with expected years to onset (*p* = 0.059, beta= 0.038).

### TNFRSF8 levels in the validation cohort

TNFRSF8 was selected for validation because, together with NfL, it was the only protein that significantly differentiated HDGECs from controls and it was also associated with symptom severity in the discovery cohort.

**Fig. 3:**
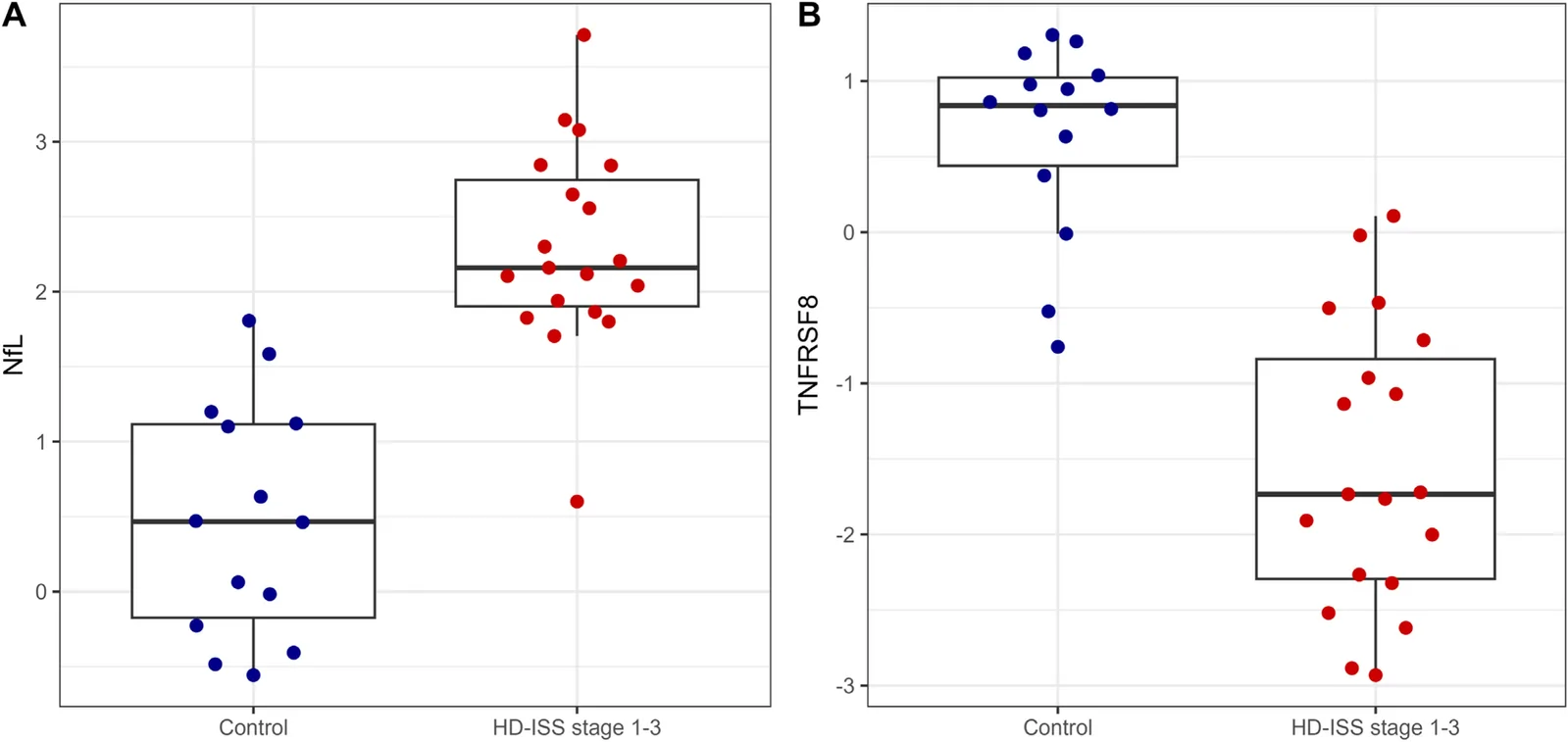
Validation cohort; NPX levels of NfL and TNFRSF8. The levels of NfL** A**, and TNFRSF8** B** were significantly altered in HD-ISS 1-3 vs. controls after adjustment for sex, age and collection site (*p* = 0.0002 and* p*= 0.001, respectively).* C* Healthy controls,* HD-ISS123* HD-Integrated Staging System stages 1-3, HDGECs,* NPX* relative protein concentration units,* a log*^2^ transformed relative measure

In the validation cohort (Fig. 3), there was also a significant reduction of TNFRSF8 levels in the HDGECs (HD-ISS 1–3) versus controls (*p*< 0.001, beta = −2.19, CI −2.78, −1.59), where the difference remained significant after adjustment for sex and age (*p* < 0.001, beta = −2.06, CI −2.75, −1.3).

As there was a difference in the level of TNFRSF8 between collection sites (Gothenburg and Stockholm), adjustment for site was included in the regression model, despite this the difference remained statistically significant (beta = −1.94, CI −3.04, −0.837, *p* = 0.001). Since TNFRSF8 was the only assay included in the statistical analysis of the validation cohort, no correction for multiplicity was conducted.

Clinical assessment in this cohort did not include all measures of the cUHDRS; however, there was a significant association with TFC (*n*= 16, spearman rho=0.65, *p* = 0.006). In summary, the results regarding TNFRSF8 in this second cohort reiterate what was found in the discovery cohort.

## Discussion

In the present study, we investigated CSF proteomic alterations in a clinically well-defined cohort of HDGECs spanning ISS stages 0–3, using a sensitive multiplex protein panel. In the discovery cohort, 16 proteins were differentially expressed in HDGECs (ISS stages 1–3) compared with controls. After stringent correction for multiple testing, adjustment for known confounders, and confirmation in an independent validation cohort, the most pronounced differences were observed for neurofilament light (NfL) [8, 9], a well-established biomarker of HD progression, followed by TNFRSF8 (tumor necrosis factor receptor superfamily member 8, also known as sCD30). TNFRSF8 displayed the largest effect size when compared to controls which remained significant after adjustment for multiple testing and confounders.

Reduced TNFRSF8 levels in CSF compared to controls were verified in the validation cohort. Remarkably, TNFRSF8 displayed the strongest association with CAG repeat length among all included proteins, but only weak associations with CAP score, and no association with age. This finding is unusual, as it indicates that this immune protein is not only associated with diagnosis but also with expansion size. Furthermore, a mixed-effects model analysis of repeated samples from 21 participants in the discovery cohort showed longitudinally decreasing levels of TNFRSF8, where subgroup analysis found statistical significance only for a decrease among manifest HDGECs. In contrast, NfL levels were unchanged over time, indicating TNFRSF8 may be more sensitive to longitudinal changes reflecting progression over a shorter time window, especially in more progressed cohorts. Reduced levels of TNFRSF8 also correlated with increased symptoms (cUHDRS) in the discovery cohort, remaining nominally significant even after adjustment for age, sex, and CAG; highlighting an independent potential to predict clinical progression. The association between reduced TNFRSF8 and disease progression was replicated in the validation cohort, as evidenced by a strong correlation with functional decline (TFC), as cUHDRS data were not available in this cohort.

Although half of the dysregulated proteins were decreased in HDGECs compared with controls, only a small fraction of proteins associated with disease progression (decline on the cUHDRS) were decreased (IFT20 and TNFRSF8). This pattern where a minority of proteins tracking disease progression are downregulated in HD has been reported previously [10, 24]. Analysis of proteins associated with years to onset resulted exclusively in significant correlation for NfL, and nominal association for eight other proteins. While TNFRSF8 did not demonstrate a statistically significant association with expected years to onset, a trend was observed that warrants further investigation in larger premanifest cohorts. Notably, the premanifest HDGECs were far from predicted clinical onset (on average, 17.9 years), a time point at which only alterations of NfL [25], proenkephalin [10, 26, 27], and mHTT [25, 28] have been reported. The dynamic course of NfL comprises early elevation but plateauing trends during the late manifest stage [29], whereas TNFRSF8 may be a slightly later feature that opposed to NfL appears to continuously track disease progression in advanced stages (HD-ISS 3). Collectively, these findings suggest that TNFRSF8 holds promise as a biomarker of progression especially in advanced disease stages of HD.

The findings of the present study extend and support those of two recent reports. The first used the same assay to find CSF proteins associated with TFC in only eight manifest HDGECs, identified TNFRSF8 as a top candidate, awaiting validation [24]. The second study combined MRI and proteomics, proposing a pathological process where initial striatal atrophy is necessary to initiate secondary immune dysregulation (indexed by decreased TNFRSF8) [30]. However, no data on clinical symptoms in relation to TNFRSF8 were reported [30]. The present study adds novelty by including neurologically unaffected controls to determine disease specificity, and including comprehensive UHDRS clinical ratings, statistical adjustment for multiplicity, longitudinal assessment, and predefined validation strategies, to characterize protein associations with clinical disease progression in HD.

Neuroinflammation is increasingly recognized as an important component of HD pathogenesis, involving complex interaction between central and peripheral immune response [31]. Mutant huntingtin is thought to influence inflammatory cells through both direct and indirect mechanism, contributing to the immune dysregulation and neurodegenerative process in HD [12]. Neuroinflammatory changes can be detected in HDGECs before clinical onset [32]. TNFRFS8 (or CD30) is a cell surface receptor mainly expressed on activated B and T lymphocytes [33]. Binding of TNFRSF8 (CD30) by its ligand promotes activation of the NF-κB signaling pathway [33], a pathway that has been linked to neurodegeneration in HD [34]. The soluble form of TNFRSF8 (sCD30) is generated through proteolytic shedding of the membrane-bound receptor [35]. While elevated levels of soluble TNFRSF8 are typically found in lymphomas and systemic inflammatory conditions [35, 36], comparatively little is known about the biological significance of reduced levels of TNFRSF8, particularly in neurodegenerative disorders. In multiple sclerosis, CSF TNFRFS8 levels were elevated in patients experiencing relapses compared with controls, but were highest among patients in remission, suggesting that increased TNFRSF8 may be associated with immune regulation in the context of autoimmune disease [37]. Therefore, the decline of TNFRSF8 (below the levels of controls) may indicate failing immune-regulatory signaling.

A few additional proteins were found to have either markedly reduced levels in HDGECs compared with controls or increased levels with symptomatic burden (cUHDRS). Although these proteins independently may not qualify as markers of disease progression, their biological functions point to pathways potentially involved in HD pathogenesis, and therefore warrant brief consideration. For instance, decreased concentrations in HDGECs were seen for MFGE8 (efferocytosis-related glycoprotein) and GPR101 (an orphan G protein-coupled receptor involved in growth-hormone regulation), both which have been implicated in immune and neurobiological processes [38, 39]. Although MFGE8 has not been directly linked to HD, its involvement in microglial phagocytosis, efferocytosis, and inflammatory responses in the CNS may indicate a potential relationship with immune-related and neurobiological processes associated with HD [40, 41]. Increased concentration of SFRP1 (Secreted frizzled-related- protein 1) correlated with cUHDRS, and has been implicated in regulation of inflammatory signaling and neuroinflammatory processes in the CNS and may thereby exacerbate neuroinflammation and axonal degeneration [42, 43]. Increased levels of WASHC3, a core component of the WASH complex, were also associated with cUHDRS. Given its role in endosomal sorting and intracellular trafficking [44], altered WASHC3 levels may reflect endosomal-lysosomal dysfunction, a well-recognized feature of neurodegenerative disorders such as HD [44, 45]. Although no direct interactions among the above-mentioned proteins have been established, their concurrent involvement suggests a potential convergence of complementary pathogenic mechanisms that may contribute to chronic neuroinflammation, cellular stress, and neurodegeneration [46, 47].

### Limitations

While PEA provides high sensitivity and scalability, the assay is restricted to predefined antibody-based panels selected by the manufacturer, without ability to detect other unexpected biomarkers. In addition, protein levels are reported as normalized protein expression NPX values, representing relative abundance rather than absolute concentration.

MRI data were not recorded and could have been useful for HD-ISS staging and proteomics–atrophy correlation [30]. Instead, NfL was used as a proxy to approximate HD-ISS stage 1 [17], rather than to provide formal HD-ISS classification.

Site differences in protein levels were noted in the validation cohort but were statistically corrected to ensure validity and confirm the main findings.

The sample size is moderate and limits the interpretation of the statistical tests especially in relation to the large number of proteins studied. To counter this challenge, data from longitudinal visits were included in the analysis. Further, the validation in a separate cohort served to strengthen the results and confirm their external validity.

## Conclusion

We identified novel disease-associated proteins in the CSF of HDGECs. Among these, TNFRSF8 emerged as a uniquely relevant immune biomarker, that tracks the clinical progression in HD, and potentially represents a mechanistic link between mutant huntingtin and progressive activation of the NF-κB signaling pathway. Investigations in larger cohorts, examining its temporal relationship with other neuroinflammatory protein signatures, as well as cell and animal model studies are warranted to map the mechanism of neuroimmune dysregulation associated with decreased TNFRSF8 and ultimately examine the potential to develop this axis as a drug target.

## Supplementary Information

Below is the link to the electronic supplementary material.

Supplementary file1 (XLSX 102 KB)

## References

[1] Paulsen JS, Long JD, Ross CA et al (2014) Prediction of manifest Huntington’s disease with clinical and imaging measures: a prospective observational study. Lancet Neurol 13(12):1193–1201. 10.1016/S1474-4422(14)70238-8 PMC4373455 25453459

[2] Ross CA, Tabrizi SJ (2011) Huntington’s disease: from molecular pathogenesis to clinical treatment. Lancet Neurol 10(1):83–98. 10.1016/S1474-4422(10)70245-3 21163446

[3] Marder K, Zhao H, Myers RH et al (2000) Rate of functional decline in Huntington’s disease. Neurology 54(2):452–452. 10.1212/WNL.54.2.452 10668713

[4] Reiner A, Dragatsis I, Dietrich P (2011) Genetics and neuropathology of Huntington’s disease. Int Rev Neurobiol 98:325–372. 10.1016/B978-0-12-381328-2.00014-6 PMC4458347 21907094

[5] Genetic Modifiers of Huntington’s Disease (GeM-HD) Consortium, Group 1, Lee JM et al (2025) Genetic modifiers of somatic expansion and clinical phenotypes in Huntington’s disease highlight shared and tissue-specific effects. Nat Genet 57(6):1426–1436. 10.1038/s41588-025-02191-5 PMC13132264 40490511

[6] Tabrizi SJ, Scahill RI, Durr A et al (2011) Biological and clinical changes in premanifest and early stage Huntington’s disease in the TRACK-HD study: the 12-month longitudinal analysis. Lancet Neurol 10(1):31–42. 10.1016/S1474-4422(10)70276-3 21130037

[7] Tabrizi SJ, Scahill RI, Owen G et al (2013) Predictors of phenotypic progression and disease onset in premanifest and early-stage Huntington’s disease in the TRACK-HD study: analysis of 36-month observational data. Lancet Neurol 12(7):637–649. 10.1016/S1474-4422(13)70088-7 23664844

[8] Byrne LM, Rodrigues FB, Blennow K et al (2017) Neurofilament light protein in blood as a potential biomarker of neurodegeneration in Huntington’s disease: a retrospective cohort analysis. Lancet Neurol 16(8):601–609. 10.1016/S1474-4422(17)30124-2 PMC5507767 28601473

[9] Niemelä V, Landtblom AM, Blennow K, Sundblom J (2017) Tau or neurofilament light—which is the more suitable biomarker for Huntington’s disease? PLoS ONE 12(2):e0172762. 10.1371/journal.pone.0172762 PMC5328385 28241046

[10] Niemela V, Landtblom A, Nyholm D et al (2021) Proenkephalin decreases in cerebrospinal fluid with symptom progression of Huntington’s disease. Mov Disord 36(2):481–491. 10.1002/mds.28391 PMC7984171 33247616

[11] Farag M, Murphy MJ, Hobbs NZ et al (2026) Cerebrospinal fluid proenkephalin predicts striatal atrophy decades before clinical motor diagnosis in Huntington’s disease. Mov Disord 41(1):95–106. 10.1002/mds.70062 PMC12882043 41000014

[12] Crotti A, Glass CK (2015) The choreography of neuroinflammation in Huntington’s disease. Trends Immunol 36(6):364–373. 10.1016/j.it.2015.04.007 PMC4786070 26001312

[13] Rodrigues FB, Byrne LM, McColgan P et al (2016) Cerebrospinal fluid inflammatory biomarkers reflect clinical severity in Huntington’s disease. PLoS ONE 11(9):e0163479. 10.1371/journal.pone.0163479 PMC5033331 27657730

[14] Petrera A, Von Toerne C, Behler J et al (2021) Multiplatform approach for plasma proteomics: complementarity of Olink proximity extension assay technology to mass spectrometry-based protein profiling. J Proteome Res 20(1):751–762. 10.1021/acs.jproteome.0c00641 33253581

[15] Snell C, Smith J, Castleton A et al (2020) How well does the composite unified Huntington’s disease rating scale (cUHDRS) reflect disease progression in Huntington’s disease (HD)? *Mov Disord*. 35. https://www.mdsabstracts.org/abstract/how-well-does-the-composite-unified-huntingtons-disease-rating-scale-cuhdrs-reflect-disease-progression-in-huntingtons-disease-hd/. Accessed December 15, 2025.

[16] Tabrizi SJ, Schobel S, Gantman EC et al (2022) A biological classification of Huntington’s disease: the Integrated Staging System. Lancet Neurol 21(7):632–644. 10.1016/S1474-4422(22)00120-X 35716693

[17] Pérez-Pérez J, Olmedo-Saura G, González RP, Pdf S (2026) Neurofilament light chain and mutant Huntingtin across HD-ISS in Huntington’s disease: a cross-sectional analysis. SSRN. 10.2139/ssrn.6068271 42876089

[18] Parkin GM, Thomas EA, Corey-Bloom J (2023) Plasma NfL as a prognostic biomarker for enriching HD-ISS stage 1 categorisation: a cross-sectional study. EBioMedicine 93:104646. 10.1016/j.ebiom.2023.104646 PMC10363447 37315450

[19] Zhang Y, Long JD, Mills JA et al (2011) Indexing disease progression at study entry with individuals at‐risk for Huntington disease. Am J Med Genet B Neuropsychiatr Genet 156(7):751–763. 10.1002/ajmg.b.31232 PMC3174494 21858921

[20] Warner JH, Long JD, Mills JA et al (2022) Standardizing the CAP score in Huntington’s disease by predicting age-at-onset. J Huntingt Dis 11(2):153–171. 10.3233/JHD-210475 35466943

[21] Wik L, Nordberg N, Broberg J et al (2021) Proximity extension assay in combination with next-generation sequencing for high-throughput proteome-wide analysis. Mol Cell Proteomics 20:100168. 10.1016/j.mcpro.2021.100168 PMC8633680 34715355

[22] Kuhle J, Barro C, Andreasson U et al (2016) Comparison of three analytical platforms for quantification of the neurofilament light chain in blood samples: ELISA, electrochemiluminescence immunoassay and Simoa. Clin Chem Lab Med (CCLM) 54(10):1655–1661. 10.1515/cclm-2015-1195 27071153

[23] Langbehn D, Brinkman R, Falush D, Paulsen J, Hayden M (2004) A new model for prediction of the age of onset and penetrance for Huntington’s disease based on CAG length. Clin Genet 65(4):267–277. 10.1111/j.1399-0004.2004.00241.x 15025718

[24] McGarry A, Moaddel R (2025) A pilot proteomic analysis of Huntington’s disease by functional capacity. Brain Sci 15(1):76. 10.3390/brainsci15010076 PMC11764106 39851443

[25] Scahill RI, Zeun P, Osborne-Crowley K et al (2020) Biological and clinical characteristics of gene carriers far from predicted onset in the Huntington’s disease young adult study (HD-YAS): a cross-sectional analysis. Lancet Neurol 19(6):502–512. 10.1016/S1474-4422(20)30143-5 PMC7254065 32470422

[26] Barschke P, Abu-Rumeileh S, Al Shweiki MHDR et al (2022) Cerebrospinal fluid levels of proenkephalin and prodynorphin are differentially altered in Huntington’s and Parkinson’s disease. J Neurol 269(9):5136–5143. 10.1007/s00415-022-11187-8 PMC9363351 35737109

[27] Scahill RI, Farag M, Murphy MJ et al (2025) Somatic CAG repeat expansion in blood associates with biomarkers of neurodegeneration in Huntington’s disease decades before clinical motor diagnosis. Nat Med 31(3):807–818. 10.1038/s41591-024-03424-6 PMC11922752 39825149

[28] Wild EJ, Boggio R, Langbehn D et al (2015) Quantification of mutant huntingtin protein in cerebrospinal fluid from Huntington’s disease patients. J Clin Invest 125(5):1979–1986. 10.1172/JCI80743 PMC4463213 25844897

[29] Rodrigues FB, Byrne LM, Tortelli R et al (2020) Mutant huntingtin and neurofilament light have distinct longitudinal dynamics in Huntington’s disease. Sci Transl Med 12(574):eabc2888. 10.1126/scitranslmed.abc2888 PMC7611886 33328328

[30] Bockholt HJ, Clemsen JD, Baker BT, Calhoun VD, Paulsen JS (2026) A two-track model of huntington’s disease pathology: striatal atrophy mediates maladaptive immune dysregulation. Int J Mol Sci 27(5):2384. 10.3390/ijms27052384 PMC12985888 41828602

[31] Blusch A, Björkqvist M (2025) Neuroinflammation in Huntington’s disease: causes, consequences, and treatment strategies. J Huntingt Dis 14(3):258–269. 10.1177/18796397251338207 PMC12332225 40772423

[32] Björkqvist M, Wild EJ, Thiele J et al (2008) A novel pathogenic pathway of immune activation detectable before clinical onset in Huntington’s disease. J Exp Med 205(8):1869–1877. 10.1084/jem.20080178 PMC2525598 18625748

[33] Buchan SL, Al-Shamkhani A (2012) Distinct motifs in the intracellular domain of human CD30 differentially activate canonical and alternative transcription factor NF-κB signaling. PLoS ONE 7(9):e45244. 10.1371/journal.pone.0045244 PMC3445475 23028875

[34] Hansen HP, Dietrich S, Kisseleva T et al (2000) CD30 shedding from Karpas 299 lymphoma cells is mediated by TNF-α-converting enzyme. J Immunol 165(12):6703–6709. 10.4049/jimmunol.165.12.6703 11120787

[35] Nadali G, Vinante F, Stein H et al (1995) Serum levels of the soluble form of CD30 molecule as a tumor marker in CD30+ anaplastic large-cell lymphoma. J Clin Oncol 13(6):1355–1360. 10.1200/JCO.1995.13.6.1355 7751879

[36] Ahn ER, Podack ER, Lander G, Bidot CJ, Jy W, Ahn YS (2004) Soluble CD30 levels are highest in autoimmune hemolytic anemia (AIHA) and Lymphoma among the hematologic disorders, but are not elevated in ITP. Blood 104(11):3872–3872. 10.1182/blood.V104.11.3872.3872

[37] McMillan SA, McDonnell GV, Douglas JP, Droogan AG, Hawkins SA (1998) Elevated serum and CSF levels of soluble CD30 during clinical remission in multiple sclerosis. Neurology 51(4):1156–1160. 10.1212/WNL.51.4.1156 9781547

[38] Yang Z, Wang JY, Yang F et al (2024) Structure of GPR101–Gs enables identification of ligands with rejuvenating potential. Nat Chem Biol 20(4):484–492. 10.1038/s41589-023-01456-6 37945893

[39] Howangyin KY, Zlatanova I, Pinto C et al (2016) Myeloid-epithelial-reproductive receptor tyrosine kinase and milk fat globule epidermal growth factor 8 coordinately improve remodeling after myocardial infarction via local delivery of vascular endothelial growth factor. Circulation 133(9):826–839. 10.1161/CIRCULATIONAHA.115.020857 PMC4767109 26819373

[40] Li D, Lu W, Wang R, Ma Y (2025) MFG‐E8 in microglial regulation: a review of basic research in the central nervous system. J Food Sci 90(7):e70235. 10.1111/1750-3841.70235 40616324

[41] Yang HM, Yang S, Huang SS, Tang BS, Guo JF (2017) Microglial activation in the pathogenesis of Huntington’s disease. Front Aging Neurosci 9:193. 10.3389/fnagi.2017.00193 PMC5474461 28674491

[42] Rueda-Carrasco J, Martin-Bermejo MJ, Pereyra G et al (2021) SFRP1 modulates astrocyte-to-microglia crosstalk in acute and chronic neuroinflammation. EMBO Rep 22(11):e51696. 10.15252/embr.202051696 PMC8567217 34569685

[43] Yao X, Kong L, Qiao Y et al (2024) Schwann cell-secreted frizzled-related protein 1 dictates neuroinflammation and peripheral nerve degeneration after neurotrauma. Cell Rep Med 5(11):101791. 10.1016/j.xcrm.2024.101791 PMC11604536 39426375

[44] Courtland JL, Bradshaw TW, Waitt G et al (2021) Genetic disruption of WASHC4 drives endo-lysosomal dysfunction and cognitive-movement impairments in mice and humans. Elife 10:e61590. 10.7554/eLife.61590 PMC7984842 33749590

[45] Federspiel JD, Greco TM, Lum KK, Cristea IM (2019) Hdac4 interactions in Huntington’s disease viewed through the prism of multiomics. Mol Cell Proteomics 18(8):S92–S113. 10.1074/mcp.RA118.001253 PMC6692770 31040226

[46] Rueda‐Carrasco J, Martin‐Bermejo MJ, Pereyra G et al (2021) SFRP1 modulates astrocyte‐to‐microglia crosstalk in acute and chronic neuroinflammation. EMBO Rep 22(11):EMBR202051696. 10.15252/embr.202051696 PMC8567217 34569685

[47] Watts TH, Yeung KKM, Yu T, Lee S, Eshraghisamani R (2025) TNF/TNFR superfamily members in costimulation of T cell responses—revisited. Annu Rev Immunol 43(1):113–142. 10.1146/annurev-immunol-082423-040557 39745933

